# Advancing the Understanding of Oral Health Through Multidisciplinary and Translational Perspectives Insights from a Special Issue of the *Journal of Clinical Medicine*

**DOI:** 10.3390/jcm14176050

**Published:** 2025-08-27

**Authors:** José João Mendes

**Affiliations:** Egas Moniz Center for Interdisciplinary Research (CiiEM), Egas Moniz School of Health and Science, 2829-511 Almada, Portugal; jmendes@egasmoniz.edu.pt

Oral health has increasingly been recognized as a critical component of overall health and well-being [1,2,3]. The complex interplay between oral conditions and systemic diseases [4,5,6] has prompted a surge in interdisciplinary research, diagnostic innovation, and preventive strategies [7,8,9]. This Special Issue of the *Journal of Clinical Medicine* brings together fourteen original studies that reflect the breadth and depth of contemporary oral health research, emphasizing translational approaches, patient-centered care, and interdisciplinary collaboration.

**Expanding Knowledge on Oral–Systemic Interactions**

Multiple studies in this collection emphasize the bidirectional relationship between oral and systemic health. Research examining inflammatory and salivary biomarkers highlights the diagnostic potential of saliva in systemic conditions, particularly in populations with neurodegenerative or metabolic disorders [10]. Additionally, a twelve-year longitudinal study examining an interdisciplinary care model for children with nephrotic syndrome demonstrates substantial improvements in oral hygiene, reduced active caries, and better gingival health [11]. A retrospective study showed that tooth wear is prevalent and increases with age, underscoring the necessity for timely and accurate diagnosis to minimize its progression [12]. Furthermore, a study in people living with diabetes in Romania showed that this population face oral health challenges and care barriers, emphasizing the need for preventive strategies, oral health education, and integrated diabetes care [13].

These findings underscore how integrated care can effectively reduce oral inflammation and contribute to systemic health stability, alongside the previously demonstrated importance of oral inflammation maintenance [14].

**Innovations in Diagnosis and Clinical Assessment**

Advancements in diagnostic accuracy are evident in studies exploring physiological parameters and their clinical implications [15]. The correlation between salivary pH and erosive tooth wear, particularly in young adults, reinforces the protective role of saliva and highlights the need for early-monitoring strategies [16]. Another study comparing cephalometric measurements evaluates newer angular parameters such as Tau and Yen angles, which show potential for improving orthodontic diagnosis [17]. These investigations underscore the utility of objective, reproducible clinical tools in both everyday practice and academic training. Furthermore, this Special Issue includes two comprehensive reviews addressing the role and precision of virtual articulators [18] and advancements in diagnostic and imaging technologies in dentistry [19].

Another important contribution in this section investigated the relationship between salivary lactate dehydrogenase (LD) levels and periodontal inflammation [20]. Using a novel test kit, researchers measured LD levels in resting saliva samples from a cohort of 110 patients and assessed their association with periodontal inflamed surface area (PISA) and its Japanese-adapted version. The findings demonstrated a strong positive correlation between salivary LD levels and both PISA indices, supporting the feasibility of this rapid and non-invasive method for periodontal screening. Moreover, the development of a predictive formula incorporating LD level, sex, and age opens the door to more accessible and scalable assessment tools for periodontal disease monitoring [20].

**Focusing on Pediatric and Developmental Oral Health**

Oral health during childhood sets the stage for lifelong well-being [21]. The validation of the COHIP-SF19 instrument into Portuguese offers a culturally adapted tool to assess oral-health-related quality of life in younger populations. Findings reveal strong internal consistency and discriminative validity, supporting its relevance for both clinical and public health applications [22]. An umbrella review on the use of conscious sedation in pediatric dentistry further reinforces the need for higher-quality evidence to support clinical protocols [23]. In addition, another umbrella review stressed that the lack of studies presenting evidence on the topic of poor dental hygiene in children with autism spectrum disorder and high parental stress levels [24].

**Therapeutic Interventions and Quality of Life**

The impact of treatment modalities on patients’ quality of life is addressed in several contributions. One study evaluating implant-supported overdentures reports meaningful improvements across all domains of oral-health-related quality of life compared to conventional dentures [25]. Another clinical trial testing a cannabidiol-infused mouthwash indicates reductions in gingival inflammation, suggesting the therapeutic promise of novel, naturally derived products [26]. These interventions reflect the growing emphasis on not only clinical efficacy, but also patient comfort and satisfaction [27,28].

**Cross-Cutting Themes and Future Directions**

This Special Issue collectively illustrates a set of recurring priorities: the centrality of oral-systemic links, the promise of biomarker-based diagnostics, the value of culturally validated assessment tools, and the growing emphasis on interdisciplinary collaboration. As we look ahead, several pathways merit further exploration: the integration of digital tools and artificial intelligence in diagnostics; longitudinal population studies to track oral-systemic trajectories; and policy frameworks that embed oral health into general health promotion strategies.

We extend our sincere gratitude to all contributing authors for their thoughtful and innovative work. Their collective efforts underscore the vibrant, interdisciplinary nature of oral health research and its profound implications for individual and public health.
